# Higher Caspase-like activity in symptomatic isolates of *Blastocystis* spp

**DOI:** 10.1186/1756-3305-7-219

**Published:** 2014-05-12

**Authors:** Dhurga D Balakrishnan, Suresh G Kumar

**Affiliations:** 1 Department of Parasitology, Faculty of Medicine, University of Malaya, Kuala Lumpur 50603, Malaysia

**Keywords:** *Blastocystis* spp, Apoptosis, Symptomatic, Asymptomatic, Caspase-like proteases, Programmed cell death, Metronidazole

## Abstract

**Background:**

Biochemical evidence of a caspase-like execution pathway has been demonstrated in a variety of protozoan parasites, including *Blastocystis* spp. The distinct differences in the phenotypic characterization reported previously have prompted us to compare the rate of apoptosis in *Blastocystis* spp. isolated from individuals who were symptomatic and asymptomatic. In the current study, we analysed the caspase activation involved in PCD mediated by a cytotoxic drug, (metronidazole) in both symptomatic & asymptomatic isolates.

**Methods:**

Apoptosis was induced in *Blastocystis* spp. by treating cultures of symptomatic and asymptomatic isolates of 3 sub-types namely 1, 3 and 5 with two different concentrations, 0.1 and 0.0001 mg/ml of metronidazole (with and without pre-treatment with a pan-caspase inhibitor, zVAD.fmk). The experiment was repeated to assess the number of apoptotic cells in all the isolates of both conditions.

**Results:**

Symptomatic isolates of subtype 3 (without pre-treatment with a pan-caspase inhibitor, zVAD.fmk) showed high fluorescence intensity for active caspase-like proteases [0.0001 mg/ml, 88% (p < 0.001) at 0.1 mg/ml, 70% (p < 0.001)] at the 72^nd^ hour *in vitro* culture in comparison with asymptomatic isolates [0.0001 mg/ml, 65%, at 0.1 mg/ml, 55%]. The number of apoptotic cells was higher [0.0001 mg/ml, 89% (p < 0.001) and at 0.1 mg/ml, 70% (p < 0.001)] at the 72^nd^ hour of *in vitro* culture in comparison with asymptomatic isolates [0.0001 mg/ml, 66% (p < 0.001) and at 0.1 mg/ml, 45% (p < 0.01)]. Cells treated with metronidazole in the presence of zVAD.fmk showed less than 10% caspase activation.

**Conclusion:**

The high number of symptomatic cells expressing active caspase-like proteases and becoming apoptotic compared to asymptomatic cells clearly demonstrates that the response to metronidazole treatment is isolate dependent. Hence this justifies the conflicting reports on the curative success rates when treated with this drug. The study has also created a need to identify apoptosis effectors in *Blastocystis* spp of different isolates especially as it was shown that apoptosis was sub-typed related. These findings can be exploited for the development of diagnostic markers and novel therapeutic drugs to enhance the effectiveness of the diagnosis and treatment of the patients infected with *Blastocystis* spp.

## Background

Depending on the origin of the death stimuli, apoptotic response could be mediated through the caspase-dependent and an independent (involving mitochondria) pathway. The first involves the activation of caspases (cysteine proteases that cleave after an aspartate residue in substrates). Caspases, the regular executioners of apoptosis, consist of 2 major groups, the effectors and initiators. Though both groups share similar general features, their mode of activation, inhibition and release of inhibition are differently regulated [1].

Cell death occurrences were also reported in the presence of the pan-caspase inhibitor zVAD.fmk, indicating the existence of caspase independent death effectors [2]. In such studies, this caspase-independent pathway was referred to as a mitochondrial pathway. This pathway involves mitochondrial dysregulation resulting from a loss of mitochondrial membrane potential and release of pro-apoptotic proteins leading to cell death. Mitochondrial pro-apoptotic proteins were shown to be involved in caspase independent pathways [3]. Both caspase-3-like activation and mitochondrial dysregulation have been reported to occur during apoptosis in *Blastocystis* spp. although the same study also reported that, both pathways, when blocked, could not save the parasite from death [4].

*Blastocystis*spp.is an extracellular unicellular enteric parasite with conflicting reports on its pathogenic potential. The symptoms are mainly bloating stomach, vomiting, abdominal pain, as well as mucous and watery diarrhoea [5]. Irritable bowel syndrome (IBS) [6] and dermatological disorders [7,8] are also frequently associated with blastocystosis. It is transmitted through the fecal oral route, and metronidazole, a cytotoxic drug is still the treatment of choice for *Blastocystis* infections despite its ineffectiveness against *Blastocystis* spp. being demonstrated [9-12] These conflicting findings led us to believe that, probably, the response towards metronidazole could be more isolate related involving different mechanisms in apoptosis especially as we have already previously established that apoptosis is subtype influenced [12]. This and our previous finding of phenotypic differences between *Blastocystis* spp. from symptomatic and asymptomatic patients [13] is currently exploited further to assess if there are differences in caspase-like activities in parasites isolated from symptomatic and asymptomatic isolates.

## Methods

### *In vitro* culture of parasites

A total of 7 symptomatic isolates of *Blastocystis* spp. 3 of subtype 3 (st3), 2 each of subtype 1 (st1), and subtype 5 (st5) and 7 asymptomatic isolates, 3 of subtype 3 (st3), 2 each of subtype 1 (st1), and subtype 5 (st5) respectively were obtained from separate infected individuals. Screening to rule out other bacterial, viral and protozoan pathogens was carried out to ensure that the symptoms in persons were only related to *Blastocystis*. Symptomatic patients harbouring the parasites showed diarrhoea and bloating stomach whereas asymptomatic isolates were obtained from infected persons who did not show any symptoms. The isolated parasites were maintained through *in vitro* cultivation in Jones’ medium supplemented with 10% horse serum and incubated at 37°C [14,15]. A pea sized stool amount was taken from every stool cup and introduced into culture tubes containing 3 ml of Jones’ medium. The parasites were maintained and sub-cultured once every 3 to 4 days for at least 1 month prior to this study. DNA was extracted directly from the culture samples using the QIAGEN Stool Mini Kit (QIAGEN, Hilden, Germany). Five μl of DNA preparations were used to amplify genomic sequences in 25 μl of PCR mixture. PCR amplification for each primer pair was repeated three times for each isolate. The isolates were then subjected to sequenced-tagged site (STS) primer-polymerase chain reaction (PCR) using seven sets of primers, namely, 351 bp for subtype 1, 526 bp for subtype 3 and 317 bp for subtype 5 [16].

### Induction of cell death via metronidazole

Metronidazole, 2-methyl-5nitroimidazole-1-ethanol, is a 5-nitroimidazole drug used for the treatment of anaerobic infections. Stock solutions of metronidazole (Discovery Fine Chemicals, Dorset, UK) were prepared in distilled water and further diluted to obtain the desired concentrations. Then, 1 × 10^5^ cells ml^-1^ were introduced into a micro- centrifuge containing the final concentration of 0.0001 mg/ml & 0.1 mg/ml of the drug in 1.5 ml micro centrifuge tube (Axygen Biosciences, Union City, California, USA). For caspase inhibition assays, 25 μM of pan-caspase inhibitor, zVAD.fmk (Z-Val-Ala-Asp.fluoromethylketone) (Pharmingen) was added to the culture medium 15 min before drug treatment. Micro- centrifuge tubes containing the same amount of parasites but untreated served as controls. Cells were then harvested at 12, 24, 36, 48, 60, 72, 84 and 96 hours for epifluorescence microscopy analysis for cytochemical assay & cell diameter measurement.

### Cytochemical staining method

#### FAM FLICA ™ CaspaTag CASPASE 3/7 Kit

Harvested cells were washed twice with 1 ml of PBS at pH 7.4. The cells were then pooled and centrifuged (2000 × g 5 min). Next, the cells were stained with FAM FLICA™ CaspaTag CASPASE 3/7 Kit (Chemicon) according to manufacturer’s protocol. The cells were examined using an epifluorescence microscope (Leitz, Wetzlar, Germany) under x400 magnification incident light transmission and the images were captured using an image analyser. The quantitative analysis of staining intensities of the cells were determined by calculating the percentage of cells stained with FAM FLICA™ CaspaTag CASPASE 3/7 kit and their respective fluorescent intensity in 100 randomly selected cells for each tube. The fluorescent intensity was expressed as arbitrary fluorescent units (AFU) and then reflected in the following manner: 1+, weak (dull green); 2+, medium (green); 3+, strong (bright green). Apoptotic cells fluoresce green. As apoptosis progresses, the number of cells undergoing active caspase increases. Cells in advanced stages of apoptosis appear brighter green.

### Detection of apoptotic and late apoptotic forms

The detection of apoptotic and necrotic cells was carried out using Apoptosis, Necrotic & Healthy Cells Qualification Kit (BiotiumInc, Hayward, USA). Harvested cells were washed twice with 1 ml of PBS at pH 7.4. Then, 1x binding buffer, 200 μM EthidiumHomodimer (III) (200 μM in PBS), FITC-Annexin V (250 μL in TE buffer containing 0.1% BSA and 0.1% NaN_3_ at pH 7.5) and Hoechst 33342 (500 μg/mL in PBS) were added sequentially. Samples were then observed under an epifluorescence microscope (Leitz, Wetzlar, Germany) using imageanalyser software. Results of cells undergoing apoptosis and necrosis were quantified with regard to percentage of apoptotic and necrotic cells in 100 cells.

### Viability of cells via cell counting

Each time the cells were harvested, viability of the cells were determined quantitatively via the trypan blue dye exclusion method and Neubauerhemocytometer chamber (Hausser Scientific, Horsham, Pennsylvania, USA). 10 μl of the cells were mixed thoroughly with 10 μl of the dye and allowed to stand for 5 min at 15°C to 30°C. Cells that were stained and unstained were e enumerated as non-viable and viable respectively [15].

This study was approved by the Medical Ethics Committee of the University Malaya Medical Centre (UMMC) Kuala Lumpur, Malaysia according to the Declaration of Helsinki.

### Statistical analysis

Statistical analysis was carried out using SPSS Statistics 18.0 software (SPSS Inc. Chicago, Illinois, USA). Independent Student’s *t*-test was used to assess the relationship of cells undergoing apoptosis in different subtypes. Correlation tests as appropriate were used to assess the correlation value between cells undergoing apoptosis and late stage apoptosis and number of cells in granular forms between different subtypes. *P* < 0.05 was considered statistically significant. Every experiment was conducted in triplicates.

## Results

### Cells expressing caspase-like activity

**Figure 1:**
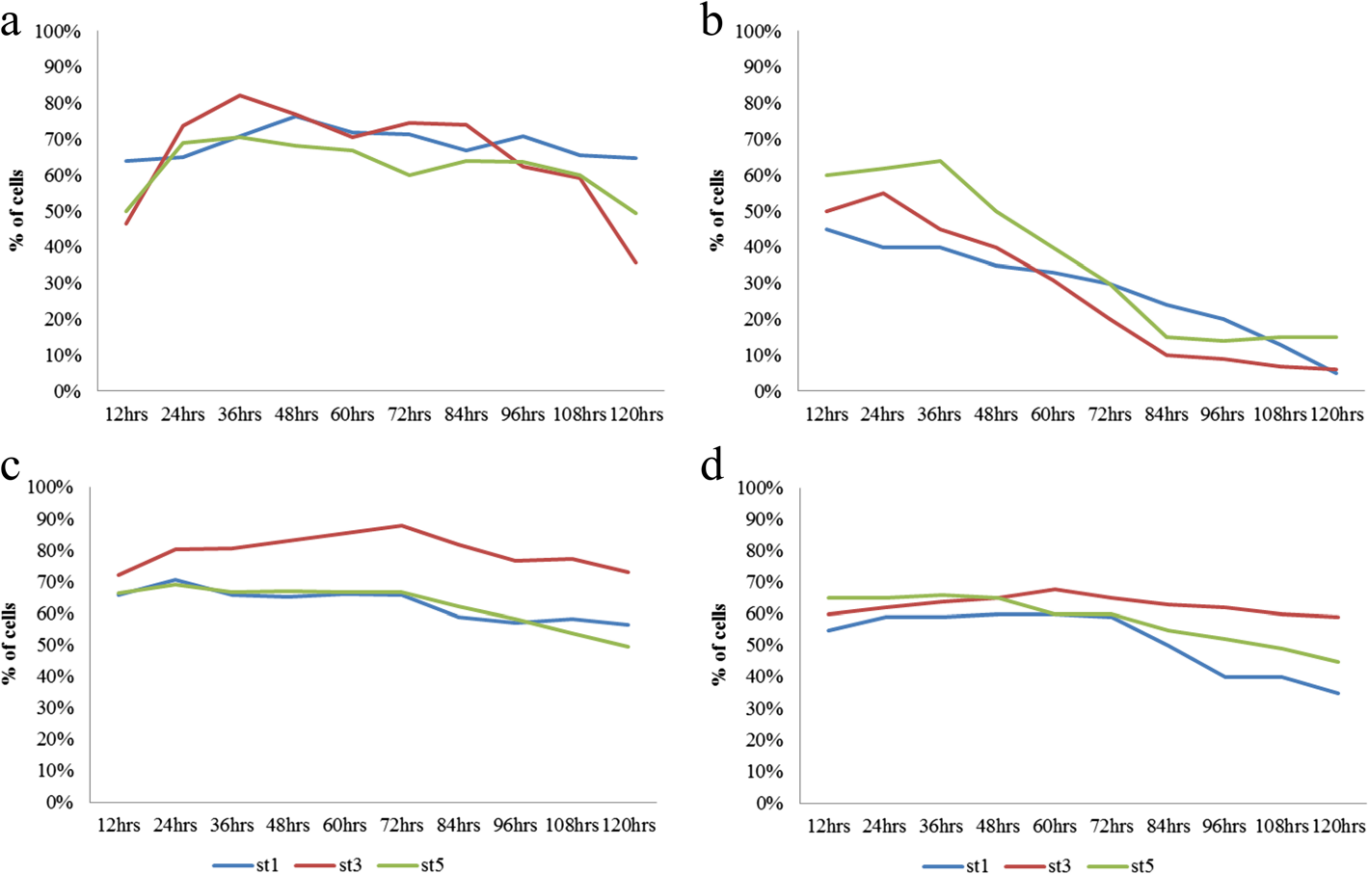
**Rate of caspase-like activity (without pre-treatment with a pan-caspase inhibitor, zVAD.fmk). (a)** untreated symptomatic **(b)** untreated asymptomatic isolates of *Blastocystis* spp. **(c)** treated (0.0001 mg/ml) symptomatic and **(d)** treated (0.0001 mg/ml) asymptomatic isolates of *Blastocystis* spp.

Caspase-like activation, an early event in apoptosis, was detected in all the symptomatic and asymptomatic isolates of both conditions (treated and untreated) [Figure 1a-d)] (without pre-treatment with a pan-caspase inhibitor, zVAD.fmk). At the 72 hr, [Figure 1c], 89% of treated symptomatic cells of subtype 3 showed significantly (p < 0.001) high fluorescence intensity for active caspase-like proteases of cells in comparison with only 75% of cells stained in untreated symptomatic subtype 3. Whereas, in the treated asymptomatic isolates [Figure 1d], 65% of the cells showed fluorescence intensity for active caspase-like proteases and 20% of apoptotic cells in the untreated asymptomatic isolates. Subtype 3 appears to have the highest number of cells expressing caspase-like activity in both symptomatic and asymptomatic isolates. But, in the untreated condition, [Figure 1a and b], number of cells that showed fluorescence intensity for active caspase-like proteases for both symptomatic and asymptomatic isolates decreased with time without significant differences between them. Hence the significance appears obvious, only after treatment, with subtype 3 showing the highest elevation of caspase-like proteases. Treated and untreated cells in the presence of pan-caspase inhibitor, zVAD.fmk showed less than 10% caspase activation (data not shown).

### Apoptotic and granular forms

**Figure 2:**
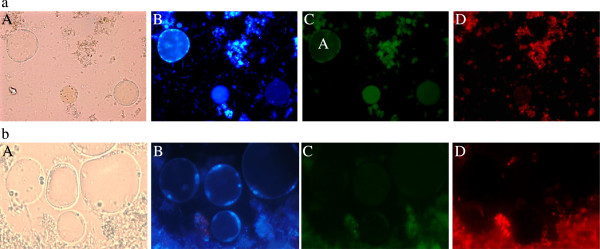
**Images of *Blastocystis *spp. stained using Hoechst stain (blue), FITC Annexin (V), and EthidiumHomodimer (III). (a)** represents Blastocystis cells from subtype 3 and 2 **(b)** represents cells from subtype 1 **(A)** light microscopy images showing drug treated (0.0001 mg/ml) *Blastocystis*spp. **(B)***Blastocystis*spp stained with Hoechst stain (blue) **(C)***Blastocystis*spp stained with Annexin (V) (green) **(D)***Blastocystis*spp stained with EthidiumHomodimer (III) (red). Annexin V labeled with fluorescein (FITC) identify apoptotic cells by binding to PS exposed on the outer membrane of *Blastocystis* spp. EthD-III is a highly positively charged nucleic acid probe, which is impermeant to live cells or apoptotic cells but stains late apoptotic-necrotic cells. (A: Apoptotic).

All the cells that were apoptotic appeared granular under bright field [Figure 2 (a & b)-A] and stained positive for apoptosis using FITC Annexin (V) [Figure 2 (a & b)-C]. Symptomatic isolates of subtype 3 showed a higher number of granular forms at the 72^nd^ hour of *in vitro* culture in comparison with other asymptomatic isolates at the same hour namely subtypes 1, 3 and 5 where at 0.0001 mg/ml, 81% (p < 0.001) and at 0.1 mg/ml, 70% (p < 0.01) of cells were granular. Only 68% of cells were apoptotic and granular in subtype 1, 65% in subtype 3 and 60% in subtype 5.

### Apoptotic and viable forms

**Figure 3:**
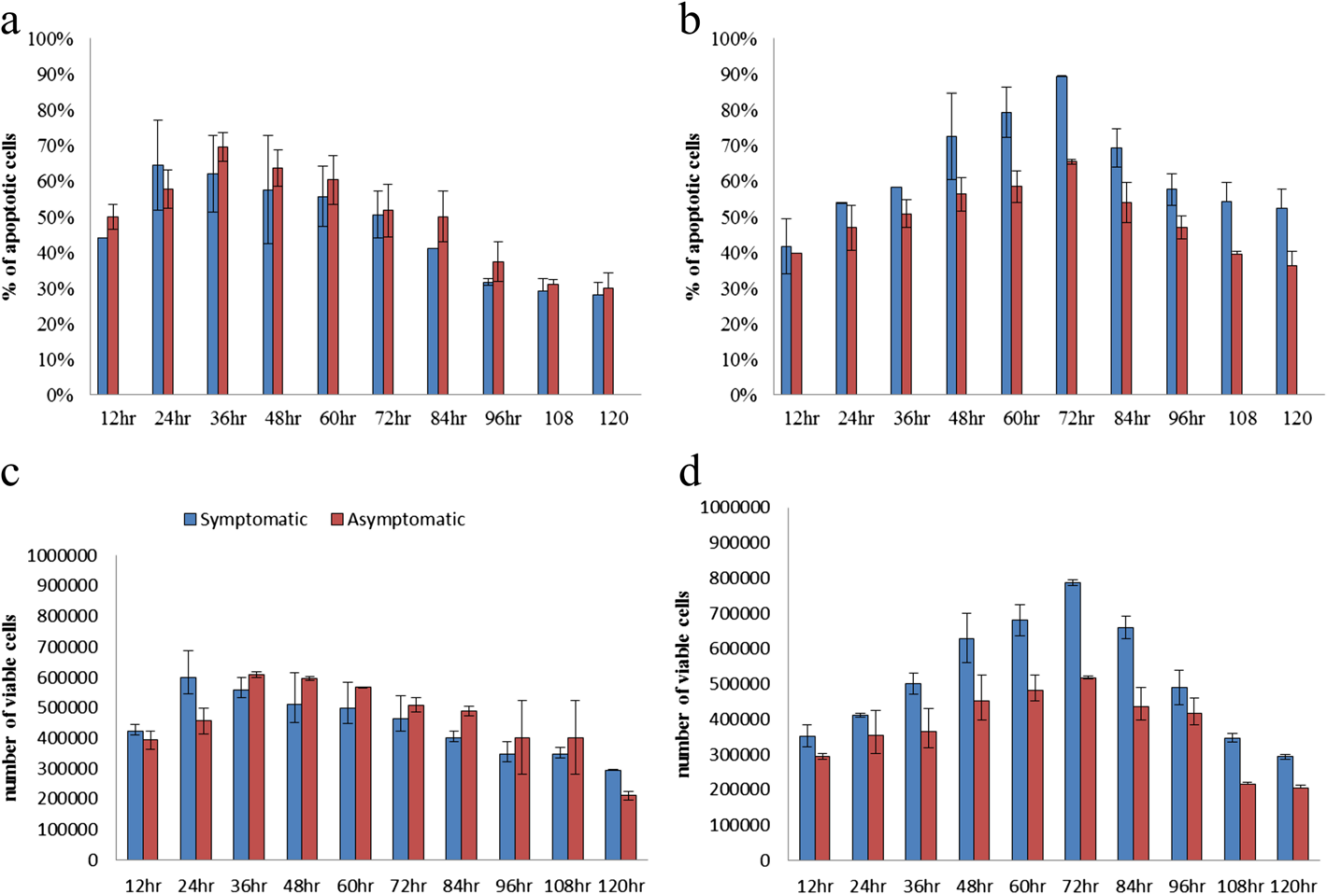
**Apoptotic and viability rate of both treated and untreated *Blastocystis *spp.** (Without pre-treatment with a pan-caspase inhibitor, zVAD.fmk) **(a)** apoptotic rate of untreated forms **(b)** treated forms (0.0001 mg/ml) of *Blastocystis*spp. **(c)** viability rate of untreated forms and **(d)** treated forms (0.0001 mg/ml) of *Blastocystis*spp.

In the untreated condition [Figure 3a] the number of apoptotic cells (stained blue and green) in the asymptomatic isolates was significantly higher (p < 0.01) in comparison with symptomatic isolates. High significant elevation of apoptotic forms [Figure 3b] were observed at the 72^nd^ hour in the symptomatic isolates in comparison with asymptomatic ones in the treated condition. Both dosages of drug show significant (p < 0.001 and p < 0.01) results in the apoptosis rate and the number of viable forms of the parasite. The number of viable apoptotic cells of the symptomatic isolates was higher in the treated condition [Figure 3d] than in the untreated condition [Figure 3c]. There is a clear correlation between viable and apoptotic cells of the symptomatic isolates. Drug treated cells showed a high correlation value (r = 0.932) for cells treated with 0.0001 mg/ml and (r = 0.899) cells treated with 0.1 mg/ml of the drug. Whereas, in asymptomatic isolates, the correlation value for cells treated with 0.0001 mg/ml (r = 0.799) and (r = 0.789) for 0.1 mg/ml of the drug. However, there was no significant correlation obtained between viable and apoptotic cells of the untreated symptomatic and asymptomatic isolates.

## Discussion and conclusion

We have reported in the past that treated *Blastocystis* spp. showed a significant decrease in diameter, in comparison with the untreated isolates [12]. In the intrinsic pathway of apoptosis), mitochondrial membranes will be depolarised causing the release of cytochrome c into the cell cytosol. Procaspase-9 will be activated downstream of mitochondrial proapoptotic events during apoptosome [17]. Dimerization of procaspase-9 molecules at Apaf-1 scaffold causes activation of caspase-9 [18]. Once initiator activated, they proteolytically activate the effector procaspases-3, -6, and -7 which subsequently cleave a specific set of protein substrates, including procaspases themselves, resulting in the start-up and amplification of the death signal. Execution of cell death will then take place with all the morphological and biochemical features usually observed [19]. The present study despite using a broad spectrum caspase inhibitor, zVAD.fmk, still showed caspase –like activity demonstrating possible involvement of uncharacterized caspase-like proteases unaffected by the pan-caspase inhibitor, as previously reported by Nasirudeen [20] and the possible role of for mitochondria in *Blastocystis* spp as many studies have reported that mitochondrial dysregulation could trigger caspase-independent apoptosis [4].

The occurrences of programmed cell death without the presence of caspases have been reported in few recent studies [21]. Caspase-independent cell death is usually linked to paraptosis, authophagy, or non-lysosomal cell death [21]. Moreover, MOMP (mitochondria membrane potential) controlled by Bcl-2 family proteins resides at the heart of several alternative cell death pathways [22]. Even though, more reports of non-classical apoptosis are emerging today, ours is the first study to look at the rate of caspase dependent and independent pathways involving symptomatic and asymptomatic pathways. High fluorescence intensity for active caspase-like proteases of cells in the symptomatic isolates, especially of those belonging to subtype 3, possess a higher sensitivity to metronidazole triggering a possible underlying mechanism when exposed to the drug. Metronidazole (inactivated form) enters into the mitochondria of cells through passive diffusion. The selective cytotoxicity of metronidazole relies on biochemical properties of pathogens that are lacking in aerobic cells of eukaryotic hosts harbouring them. Conversion of cells from the inactive to active form leads to further uptake of the inactive form. Thus, the rate of intracellular activation is important as it affects drug uptake via a concentration gradient [23]. Once inside, the drug competes with the natural electron transport system of the organism. The organism then transfers electrons to the nitro group of the drug [24]. This reduction causes the drug to release cytotoxic radicals, thus killing the cell [24]. This raises the possibility that the drug sensitivity could be more enhanced in symptomatic isolates due to the higher rate of intracellular activation in the symptomatic isolates, thus resulting in an increased uptake of drug before transferring their own electrons to the nitro group of the drug. This could have increased the rate of apoptosis in the symptomatic isolates.

Untreated isolates of both the symptomatic and asymptomatic group, too, underwent apoptosis but at a lower rate compared to treated cells. This is because in normal cultures, especially after sub-culture, parasites do tend to be apoptotic naturally as the process of cell death is an intrinsic part of the parasite’s life cycle.

*Blastocystis* spp. in symptomatic patients could have made infected individuals seek treatment. It is uncertain whether such treatment significantly altered the biochemical aspects of the parasite which in turn when exposed to metronidazole in the present study could have caused higher sensitization of the caspase pathway. In this study, despite inhibiting the caspase pathways the subtype 3 of the symptomatic isolates showed the highest number of cells becoming apoptotic in comparison with the asymptomatic isolates.

Previously we have shown that symptomatic isolates have a lower growth rate compared to asymptomatic isolates which is probably attributed to the higher apoptotic activity in the symptomatic isolates [13].

The present study also showed that drug induced cells which are apoptotic were observed to be granular in nature concurring with the observation by Haresh [25] that the treated parasites could have reverted to granular forms as a survival mechanism to release reproductive granules and thus increase the number of parasites in cultures. It is highly likely that the initial stage of apoptosis seen by the high caspase-like activity triggers a regulatory mechanism of survival for the cells to be granular. Hence this justifies the correlation between apoptotic and the numbers of viable cells providing evidence that the process of apoptosis in *Blastocystis* spp., while leading to cell death could also trigger a regulatory mechanism for cell survival. Upon drug exposure, the cell effectors (already activated by apoptotic receptors) could have signalled the reproduction mechanism to produce more granules. Most probably, it is the cell number of the parasite that them pathogenic. Perhaps this is why, as the apoptosis rate increases, the cells which are becoming viable in symptomatic isolates are significantly higher in comparison with isolates from the asymptomatic ones. Thus, as reported previously, cell death mechanisms were allegedly adapted over time, congruent not with their emergence, but, their regulation. The present study justifies what Taylor-Brown and Hurd [26] recently stated; that there is no such thing as a *bona fide* cell death programme. It is most likely that, from the early stage of cellular life, that both the cell differentiation and cell cycle processes had intrinsic error rates that would have needed regulation to avoid destruction of the cell [26]. Ameisen [27] continued with this idea, by suggesting that the genes involved with the cell processes would have evolved in conjunction with the genes associated with error rate-limiting control mechanisms. In fact, there have been many studies reporting that most genes involved in metazoan cell death programmes can be seen to have other primary functions. Protozoa lacks most of the molecules involved in apoptotic death of multicellular organisms [28]. The presence of caspase-like protease indicates that there are some molecular similarities between apoptosis of higher animals and protozoans. This warrants further research on the apoptotic machinery of the parasitic protozoa. The need for elucidating the association between the mode of the drug and the biochemical pathways involved in apoptosis or non-classical apoptosis after drug exposure needs to be exploited for the development of novel therapeutic drugs and diagnostic markers.

## Competing interests

The authors declare that they have no competing interests.

## Author’s contributions

KGS and DBD were involved in the intellectual planning of the study. DBD and KGS were involved in designing the study. Experiments were carried out by DBD. Analysing the data and manuscript preparation were done by DBD and KGS. KGS and DBD edited the paper. Both authors read and approved the final manuscript. KGS is the guarantor of the paper.
